# The Effects of High-Intensity Interval Exercise and Hypoxia on Cognition in Sedentary Young Adults

**DOI:** 10.3390/medicina55020043

**Published:** 2019-02-10

**Authors:** Shengyan Sun, Paul D. Loprinzi, Hongwei Guan, Liye Zou, Zhaowei Kong, Yang Hu, Qingde Shi, Jinlei Nie

**Affiliations:** 1 Faculty of Education, University of Macau, Macao, China; sysun@zjhu.edu.cn (S.S.); zwkong@um.edu.mo (Z.K.); 2 Department of Physical Education, Huzhou University, Huzhou 313000, China; 3 Department of Health, Exercise Science and Recreation Management, School of Applied Sciences, The University of Mississippi, Oxford, MS 38677, USA; pdloprin@olemiss.edu; 4 Department of Health Promotion and Physical Education, School of Health Sciences and Human Performance, Ithaca College, Ithaca, NY 14850, USA; hguan@ithaca.edu; 5 Lifestyle (Mind-Body Movement) Research Center, College of Sports Science, Shenzhen University, Shenzhen 518060, China; 6 Sports Science Research Center, Beijing Sport University, Beijing 100084, China; 7 School of Physical Education and Sports, Macao Polytechnic Institute, Macao, China; qdshi@ipm.edu.mo (Q.S.); jnie@ipm.edu.mo (J.N.)

**Keywords:** cognitive function, Go/No-Go task, reaction time, response accuracy, peripheral oxygen saturation

## Abstract

*Background and Objectives:* Limited research has evaluated the effects of acute exercise on cognition under different conditions of inspired oxygenation. Thus, the purpose of this study was to examine the effects of high-intensity interval exercise (HIE) under normoxia (inspired fraction of oxygen (FIO_2_): 0.209) and moderate hypoxia (FIO_2_: 0.154) on cognitive function. *Design:* A single-blinded cross-over design was used to observe the main effects of exercise and oxygen level, and interaction effects on cognitive task performance. *Methods:* Twenty inactive adults (10 males and 10 females, 19–27 years old) performed a cognitive task (i.e., the Go/No-Go task) before and immediately after an acute bout of HIE under normoxic and hypoxic conditions. The HIE comprised 10 repetitions of 6 s high-intensity cycling against 7.5% body weight interspersed with 30 s passive recovery. Heart rate, peripheral oxygen saturation (SpO_2_) and rating of perceived exertion were monitored. *Results:* The acute bout of HIE did not affect the reaction time (*p* = 0.204, η^2^ = 0.083) but the accuracy rate decreased significantly after HIE under both normoxic and hypoxic conditions (*p* = 0.001, η^2^ = 0.467). Moreover, moderate hypoxia had no influence either on reaction time (*p* = 0.782, η^2^ = 0.004) or response accuracy (*p* = 0.972, η^2^ < 0.001). *Conclusions:* These results indicate that an acute session of HIE may impair response accuracy immediately post-HIE, without sacrificing reaction time. Meanwhile moderate hypoxia was found to have no adverse effect on cognitive function in inactive young adults, at least in the present study.

## 1. Introduction

Moderate-intensity level exercise or physical activity is generally accepted to have positive effects on cognitive function [1], among both young adults and older adults with cognitive impairment [2,3]. A single bout of moderate-intensity aerobic running (15 min) appears to improve some selected aspects (e.g., executive function, cognitive processing speed, selective attention, visual-spatial ability, and verbal memory) of cognitive performance in young healthy adults [4,5,6]. Longitudinal aerobic interventions have also been shown to reverse age-related brain volume decline and improve memory in older adults [6,7]. Peripheral levels of catecholamine and dopamine [8], as well as neuronal growth factors (e.g., brain-derived neurotrophic factor, insulin-like growth factor) [7,8,9] are reported to increase following a moderate exercise training regime. The exercise-induced upregulation of these neurotransmitters and neurotrophins can facilitate cerebral neural activation and neurogenesis, and thus, result in better cognitive performance.

Much evidence has emerged in the past decade illustrating that high intensity interval exercise (HIE), an exercise modality comprising repeated bursts of vigorous exercise with less intense or passive recovery periods [10], is an effective and time-efficient way to improve many crucial indicators relating to cardio-metabolic health, such as cardiorespiratory fitness, insulin sensitivity, and body composition [10,11,12]. Although HIE has shown promising potential in improving cardiovascular health, the question of whether HIE could also improve cognitive function and brain health is just beginning to be addressed, with inconclusive findings. Some studies have reported that acute HIE can improve lexical learning [10], selective attention [13], and executive function [14] in adults, and that such beneficial effects are greater [8] and last longer [14] when compared to those resulting from continuous aerobic exercise. In contrast, an inverted U-shaped relationship between exercise intensity and cognitive function has been proposed, indicating that exercise intensity higher than the anaerobic threshold (approximately 50–60% of VO_2max_ in untrained individuals) can attenuate cognitive performance [15]. Further, this intensity-specific effect may also be influenced by the temporality of exercise and the cognitive type being evaluated [16]. Thus, more robust evidence is needed to support the positive influence of HIE on cognitive function.

Combining hypoxia with exercise training may have synergistic effects on weight loss [17] and cardiorespiratory fitness improvement in sedentary populations [18]. The additional physiological challenges imposed by hypoxic exposure appear to be an ergogenic aid in the treatment of obesity and associated comorbidities, as well as multiple other clinical diseases [19]. However, exercise under hypoxia may have negative effects on executive function [20], because the diminished oxygen availability under hypoxic conditions can decrease the arterial oxygen content and cause cerebral hypoxia [21]. Previous studies found that scores in a number of neurocognitive domains, including processing speed, memory, executive function and attention, are reduced after acute exposure to severe hypoxia at altitudes equivalent to 4500–5500 m [22,23]. Although moderate-intensity exercise under low to moderate levels of hypoxia (corresponding to altitudes of 1300 m and 2600 m) improves reaction time and response accuracy, the beneficial effects disappear in the absence of exercise [24]. Furthermore, restoration of the cognitive function impaired during hypoxia by low- to moderate-intensity exercise has also been reported [25]. These findings indicate that the increment in cognitive performance is ascribed to exercise rather than hypoxia. Nevertheless, it remains unclear how HIE under hypoxia affects cognitive function. As the commonly used hypoxic level to develop cardiorespiratory fitness in athletes [26,27] and to improve cardiometabolic risk factors in sedentary populations [17,18] is around the inspired fraction of oxygen (FIO_2_) level of 0.154, an integrated understanding of the effect of moderate hypoxia at FIO_2_ of 0.154 (equivalent to an elevation of 2500 m) and exercise on cognition might be more practical than other hypoxic levels.

Using a single-blinded cross-over design, the purpose of this study was to examine the effects of single bouts of HIE under normoxia or moderate hypoxia (FIO_2_ = 0.154) on cognitive function. We hypothesized that cognitive function would be improved following HIE. However, we anticipated that moderate hypoxic exposure would cause an adverse effect on cognitive function in comparison to normoxia.

## 2. Materials and Methods

### 2.1. Study Participants

A priori power analysis was conducted by GPower Version 3.1 to estimate the sample size. When effect size was set at a medium effect size of 30 [28,29], 20 participants would be needed to detect a significant difference in a two-way repeated-measures ANOVA with a power of 80% and a significance level of 5%. This study was conducted in accordance with the declaration of Helsinki and approved by the Ethical Committee of the University of Macau for the Use of Human and Animal Subjects in Research. Twenty physically inactive but healthy young adults (10 males and 10 females, 19–27 years old) volunteered to participate in this study (Table 1). The inclusion criteria were: residence at altitudes lower than 1000 m; no previous experience of hypoxic training; no current engagement in any structured exercise; nonsmokers; had not taken oral contraceptives or any medication during the past 6 months; and no musculoskeletal problems. Following an explanation of the purpose and constraints of the study in detail, all participants gave their written informed consent prior to participation.

### 2.2. Experimental Procedure

All subjects completed a familiarization trial and three main experimental trials, namely, a normoxic no-exercise control trial (CON), a HIE trial under normoxia (NOR) and a HIE trial under simulated hypoxia (HYP) on four separate occasions. During the familiarization trial, subjects were familiarized with the experimental procedures, practiced the cognitive task (Go/No-Go; GNG task), and undertook anthropometric assessments and a maximal graded exercise test. The three formal experimental trials were performed at the same time of day (from 19:00 to 20:00) under a well-controlled laboratory environment with stable temperature (22 ± 1 °C) and relative humidity (72 ± 5%). The order of the main trials was randomized and counterbalanced across subjects, who were unaware of the normoxia or hypoxia conditions. Seven-day periods were interspersed between trials to remove any potential residual effects.

On the experimental days, subjects performed HIE (or rest) and cognitive tasks under either normoxic (PIO_2_: 150 mmHg, FIO_2_: 0.209) or hypoxic (PIO_2_: 117 mmHg, FIO_2_: 0.154, simulating an altitude corresponding to 2500 m) conditions. The normoxic and hypoxic gas mixtures were generated using a modified gas mixing system (Altitrainer, SMTEC, Nyon, Switzerland) and supplied to subjects through tubes and a breathing mask. On the days of the main experiments, subjects arrived at the laboratory before 19:00 and put on a breathing mask at 19:00. After exposure to normoxia or hypoxia for 30 min, they performed a pre-exercise GNG task, a HIE session (or rest for the corresponding time) on an ergometer, and a post-exercise GNG task immediately after HIE under the respective experimental conditions.

### 2.3. Maximal Graded Exercise Test

A maximal graded exercise test was performed by each subject on an ergometer (Monark 894E, Vansbro, Sweden) to measure the VO_2peak_ via a breath-by-breath modular metabolic system (MetaMax 3B, Cortex Biophysik, Leipzig, Germany). The initial workload was 50 W and the power output increased by 25 W every 3 min until exhaustion. The highest VO_2_ value averaged over 30 consecutive seconds during the last stage was recorded as VO_2peak_.

### 2.4. High-Intensity Interval Exercise

The 6-min HIE protocol consisted of 10 repetitions of 6 s of high-intensity cycling bouts interspersed with 30 s of passive recovery. Subjects pedaled as fast as possible against a resistance of 7.5% of body weight during the 6 s work durations, and underwent passive recovery during the 30 s rest periods on a cycle ergometer (Monark 839E, Vansbro, Sweden). Heart rate (HR) and peripheral oxygen saturation (SpO_2_) values before and after each bout of HIE were monitored using a pulse oximeter (Radical-7 Pulse CO-Oximeter, Masimo, Irvine, CA, USA) placed on the left middle finger. Ratings of perceived exertion (RPE; 6–20 Borg scale) were recorded after the completion of each cycling repetition.

### 2.5. Cognitive Task Measurement

Cognitive performance was measured for both reaction time and response accuracy using the GNG task. A laptop with a pre-established E-Prime program was used to administer the GNG task. For one specific trial, two symbols would appear on screen in turn (i.e., a square printed in red or blue, followed with a number or a letter printed in black). If a red square appeared on screen followed by a number, or a blue square appeared on screen followed by a letter, the subjects needed to respond by pressing the “J” button with the right index finger. In contrast, if a letter showed following the red square or a number emerged following the blue square, the subjects needed to press the “F” button with the left index finger. Each stimulus remained on screen for a maximum of 2 s, or until a response was given. Following each trial, a signal of “+” would appear on screen as an inter-stimulus interval lasting for 2 s. The GNG task included 40 trials, and the number of correct responses assigned to “F” and “J” was the same. Reaction time (ms) and response accuracy (%) were recorded to measure cognitive performance. Functional imaging studies have demonstrated associations between GNG performance and activation of several brain structures, including in the prefrontal and anterior cingulate regions of the brain [30]; both structures have been implicated in inhibitory-based, executive function in humans [31]. Detailed discussion of the factor structure and construct validity of related GNG tasks can be found elsewhere [32].

### 2.6. Habitual Physical Activity Assessment

Subjects were asked to maintain their normal diet and daily routines and avoid extra exercise during the study period. Daily physical activity was monitored using a pedometer (NL-2000i, New Lifestyles, Inc., Lee’s Summit, MO, USA) on 3 days (2 days before and the day of the experiment) for each experimental trial. This pedometer has demonstrated evidence of validity [33].

### 2.7. Statistical Analyses

The PASW software (Release 22.0; IBM, New York, NY, USA) was used for statistical analyses. Before the main analyses, the Shapiro-Wilk test was conducted to confirm that the outcome variables were normally distributed. Independent samples *t*-tests were conducted to evaluate demographic differences across sex (Table 1). In order to observe the real alternations between NOR and HYP, changes in reaction time (Δ RT) and accuracy (Δ Accuracy) were calculated using the pre- and post-values obtained in the NOR or HYP trial minus the corresponding pre- and post-values obtained in the CON trial. Two-factor repeated measures ANOVA was performed to determine the main effects of exercise (pre- and post-HIE) and oxygen level (FIO_2_: 0.209 and 0.154), and interaction effects (exercise × condition) on cognitive task performance and physiological parameters (i.e., HR, RPE and SpO_2_). Tukey’s post hoc test was used to compare the group differences when there was a significant interaction effect. The effect size of partial eta squared (η^2^) was calculated to determine the effect sizes of the main and interaction effects. The effect size was considered small if η^2^ < 0.06 and large if η^2^ > 0.14. All data were expressed as mean ± standard deviation (SD), and *p* < 0.05 was taken as the level of statistical significance.

## 3. Results

### 3.1. Exercise Data and Habitual Physical Activity

An acute bout of HIE under normoxia (FIO_2_ = 0.209) and hypoxia (FIO_2_ = 0.154) showed similar peak power, average power and fatigue index (*p* > 0.05, Table 2). There were no significant differences (*p* > 0.05) in daily activity among the three trials (CON: 7205 ± 2334 steps/day, NOR: 8987 ± 3104 steps/days and HYP: 7868 ± 3128 steps/days).

### 3.2. Physiological Parameters

After the acute bout of HIE, HR (*p* < 0.001, η^2^ = 0.974) and RPE (*p* < 0.001, η^2^ = 0.953) in both the NOR and HYP trials significantly increased in the post-exercise measurements (Table 3). However, there was no main effect of oxygen condition on HR or RPE. A significant interaction effect between exercise and FIO_2_ level was found on SpO_2_ (*p* < 0.001, η^2^ = 0.450). Under the hypoxic condition, SpO_2_ was significantly lower at rest (98 ± 1 in NOR trial vs. 96 ± 3 in HYP trial, *p* < 0.05) and after exercise (98 ± 2 in NOR trial vs. 92 ± 3 in HYP trial, *p* < 0.05) when compared to the normoxic condition (Table 3).

### 3.3. Cognitive Task

**Figure 1:**
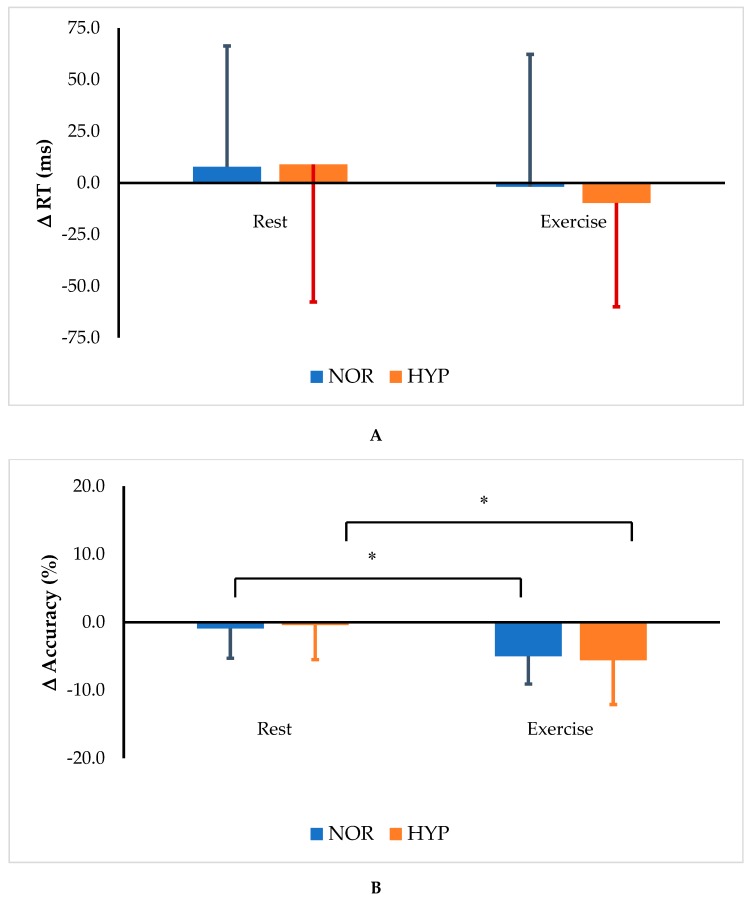
Changes in reaction time (**A**) and accuracy (**B**) subtracted from the baseline at resting at sea level on the Go/No-Go test before and after HIE under normoxia (20.9% O_2_) and hypoxia (15.2% O_2_). * *p* < 0.001 vs. Rest (pre-exercise). Tukey’s post hoc paired *t*-tests were employed.

There were no main effects of exercise (*p* = 0.204, η^2^ = 0.083) or FIO_2_ level (*p* = 0.782, η^2^ = 0.004) on the changes in reaction time (Δ RT), and no interaction between exercise and FIO_2_ level (*p* = 0.514, η^2^ = 0.023, Table 4). These findings suggested that neither HIE nor oxygen condition affect reaction time. With regard to the accuracy of the GNG task, a significant main effect of exercise on the change in accuracy (Δ accuracy) was observed (*p* < 0.001, η^2^ = 0.467). After the acute bout of HIE, Δ accuracy was significantly decreased in both the NOR and HYP trials (*p* < 0.01). In contrast, the reduction in FIO_2_ had no effect on Δ accuracy (*p* = 0.972, η^2^ = 0.000, Figure 1). These results indicated that the acute session of HIE reduced the response accuracy, but that hypoxia had no influence on this parameter.

## 4. Discussion

The current study firstly examined the effects of HIE under normoxia and moderate hypoxia on cognitive function in healthy young adults. The main findings of this study were: (1) HIE impaired cognitive function under both normoxic and hypoxic conditions, as indicated by reduced accuracy rates in post-exercise measurements; (2) moderate hypoxia (i.e., FIO_2_ = 0.154) in the present study did not affect cognitive function.

Maintaining adequate cognitive function is important for humans as it is involved in almost every aspect of daily life. There are an increasing number of studies supporting the notion that moderate physical activity can facilitate various aspects of cognitive performance [4,7,34]. However, studies regarding the impact of time-efficient HIE on cognition are notably rare. We found that the HIE protocol in the present study reduced the response accuracy of the GNG task under both normoxia and hypoxia without sacrificing the response speed. This finding, in general, aligns with past meta-analytic research showing that acute exercise may have a more beneficial effect on response speed when compared to accuracy [35]. This finding, however, conflicts with some previous studies that have shown an acute improvement in cognitive performance after high impact exercise [8,13,14]. Although the precise mechanisms responsible for the discrepancy between our study and the others are unclear, a meta-analytic review suggests that exercise intensity, subjects’ initial fitness level, the nature of the cognitive task and the timing of task administration are primary moderators in exercise–cognition interactions [36]. It appears that when the cognitive task is performed immediately following exercise, lighter intensity exercise has more positive effects, but when tested after a delayed period of time, more intense exercise triggers greater beneficial effects [36]. Our recent review and empirical work support these findings [6,16]. In line with this finding, our study failed to find positive effects on cognition as measured immediately after HIE, whereas improved cognitive performance following HIE was observed in other studies when tested after 15 min of passive [8] or active recovery [13]. In addition, the improvement in cognitive performance in response to moderate exercise observed in prior work is associated with augmented cerebral neural activation [37], which seems to be influenced by differences in exercise intensity [38]. Specifically, the arousal level and information processing in the central nervous system increased from low- to medium-intensity exercise but decreased after high-intensity exercise, presenting an inverted U-shaped relationship [15,38]. Hence, the timing of the cognitive task and the intense nature of HIE may have partly explained the reduced cognitive function observed in the present study.

The brain tissue is largely dependent on a constant and sufficient oxygen supply to function effectively [23]. In a hypoxic environment, SpO_2_, tissue saturation index and deoxyhemoglobin have been reported to decrease continually as hypoxic severity increases [21], and the consequent physiological changes, such as cerebral deoxygenation may exert detrimental effects on cognitive function [20,23]. In this study, at the beginning of each trial, cognitive performance was measured after resting under normoxic or hypoxic conditions for 30 min. SpO_2_ was decreased significantly at rest under hypoxia in comparison to normoxia, suggesting that arterial oxygen content is diminished after hypoxic exposure. But surprisingly, in contrast to our hypothesis, no differences in reaction time or response accuracy was found between the two different FIO_2_ levels. These findings indicated that the moderate hypoxia used in the present study was insufficient to adversely affect cognitive function. Moreover, when SpO_2_ was further decreased to 92 ± 3% after HIE in hypoxia, the moderate hypoxia still did not affect reaction time or accuracy rate. This finding was consistent with studies that reported no influence of moderate hypoxia (i.e., corresponding to altitudes of 1300 or 2600 m) on cognitive function [24,28], but conflicts with those that found attenuated cognitive performance during exercise under severe hypoxia (i.e., equivalent to altitudes of 3800 or 4500 m) [22,39]. It appears that the severity of hypoxia plays an important role in the exercise–cognition interaction. Cerebral and pulmonary abnormalities due to hypoxia are increasingly common from altitudes above 2500 m [40], and cognitive impairment is particularly noticeable in severe hypoxia [41]. Therefore, the FIO_2_ level of 0.154 in the present study (equivalent to an altitude of 2500 m) may be below the threshold at which hypoxia would impose distinct adverse effects on cognitive function [40]. Another possible explanation is that moderate hypoxia exposure for a duration of ~40 min is too short to provide sufficient physiological stress on the central nervous system.

It is undeniable that cerebral blood flood (CBF) and brain oxygenation may also be involved in the regulation of cognitive processes during exercise under normoxia and hypoxia through conflicting cerebral hemodynamic responses [42]. In normoxia, arterial blood pressure increases in parallel with exercise intensity. To avoid potential damage due to hyperperfusion and hypocapnia, a cerebral vasoconstriction response and subsequent reductions in CBF and brain oxygenation are observed during high-intensity exercise in normoxia [42,43]. In contrast, arterial oxygen content is decreased in hypoxia [22,24,28]. In order to compensate for the reduced oxygen availability, greater cerebral vascular conductance and increased CBF have been found to secure brain oxygen delivery in the first place [42]. The contrasting responses of cerebral vascular conductance in relation to high-intensity exercise and hypoxia may partly explain why intense HIE impaired cognitive function whereas moderate hypoxia demonstrated no influence on cognitive function in the present study. However, given that neither CBF nor brain oxygenation was measured in the present study, further studies are needed to elucidate the role of cerebral vascular conductance in the HIE–cognition interaction under moderate hypoxia.

In the present study, a relatively simple cognitive test, the GNG task, was used to measure cognitive function in relation to executive function. More sophisticated and higher-level cognitive tasks involving information processing, attention, intelligence and working memory should be included in further studies. In addition, according to previous studies [8,13], we cannot rule out the possibility that a delayed beneficial effect on cognition may be observed after recovering from intense HIE. Unfortunately, we did not determine cognitive performance following a recovery period in our study. Third, physiological responses to hypoxic HIE other than SpO_2_, including brain oxygenation and CBF were not monitored, which limited the elucidation of potential mechanisms. Finally, further interpretation of the impact of hypoxic exposure alone on cognitive function is limited because of the lack of a non-exercise hypoxic control group.

## 5. Conclusions

In summary, the present study firstly addressed the influence of a single bout of HIE under normoxia and moderate hypoxia (i.e., FIO_2_ = 0.154) on cognitive function. After HIE, the accuracy rates of the GNG task decreased under both normoxic and hypoxic conditions, suggesting that HIE may impair cognitive function immediately post-HIE. However, moderate hypoxia (i.e., equivalent to an altitude of 2500 m) was demonstrated to have no adverse effect on cognitive performance, suggesting that moderate hypoxia could be a strategy to improve cardio-metabolic health outcomes without any impairment of inhibitory-based cognitive function in sedentary adults.

## Figures and Tables

**Table 1:** The demographic characteristics across the entire sample and separately for male and female participants.

	Male		Female		All	
Age (year)	24.1	±1.4	23.6	±3.3	23.9	±2.5
Height (cm)	175.2	±6.8	159.0	±3.8 *	167.1	±9.8
Weight (kg)	69.9	±13.5	51.2	±5.0 *	60.5	±13.8
BMI (kg·m^−2^)	22.7	±3.9	20.2	±1.5	21.5	±3.2
VO_2peak_ (mL·kg^−1^·min^−1^)	36.5	±7.0	27.4	±3.9 *	31.9	±7.2

* *p* < 0.01 vs. male (independent samples *t*-test).

**Table 2:** High-intensity interval exercise (HIE) workload in normoxia and hypoxia.

	NOR		HYP	
Peak Power (w/kg)	6.8	±2.2	6.8	±2.4
Average Power (w/kg)	4.5	±0.6	4.4	±0.6
Fatigue Index (%)	45.8	±16.4	43.4	±17.7

**Table 3:** Physiological parameters pre-, during and post-exercise in normoxia and hypoxia.

					Exercise Effect		Condition Effect		Interaction Effect	
	Pre		Exe		*p Partial* η^2^		*p Partial* η^2^		*p Partial* η^2^	
HR (bpm)										
NOR	72	±6 *	138	±12	<0.001	0.974	0.085	0.156	0.275	0.066
HYP	73	±7 *	141	±12						
RPE										
NOR	6	±1 *	14	±2	<0.001	0.953	0.250	0.069	0.837	0.002
HYP	7	±1 *	14	±2						
SaO_2_ (%)										
NOR	98	±1	98	±2	<0.001	0.724	<0.001	0.550	<0.001	0.450
HYP	96	±3 *^†^	92	±3 ^†^						

* *p* < 0.001 vs. post-HIE, ^†^ *p* < 0.05 vs. normoxia. A two-factor RM-ANOVA was employed to evaluate these main and interaction effects.

**Table 4:** Changes in cognitive performance before and after HIE in normoxia and hypoxia.

					Exercise Effect		Condition Effect		Interaction Effect	
	Pre-CON		Exe-CON		*p Partial* η^2^		*p Partial* η^2^		*p Partial* η^2^	
Δ RT (ms)										
NOR	7.8	±58.5	−1.8	±64.0	0.204	0.083	0.782	0.004	0.514	0.023
HYP	9.0	±66.7	−9.7	±50.3						
Δ Accuracy (%)										
NOR	−0.9	±4.4 *	−5.0	±4.1	0.001	0.467	0.972	0.000	0.537	0.020
HYP	−0.4	±5.1 *	−5.6	±6.5						

* *p* < 0.01 vs. post-HIE; Pre-CON = pretest minus control trial; Exe-CON = Exercise condition minus control trial. A two-factor RM-ANOVA was employed to evaluate these main and interaction effects.
